# Environmental Sustainability Impacts of Solid Waste Management Practices in the Global South

**DOI:** 10.3390/ijerph191912717

**Published:** 2022-10-05

**Authors:** Ismaila Rimi Abubakar, Khandoker M. Maniruzzaman, Umar Lawal Dano, Faez S. AlShihri, Maher S. AlShammari, Sayed Mohammed S. Ahmed, Wadee Ahmed Ghanem Al-Gehlani, Tareq I. Alrawaf

**Affiliations:** 1 College of Architecture and Planning, Imam Abdulrahman Bin Faisal University, Dammam 31441, Saudi Arabia; 2 Department of Urban and Regional Planning, College of Architecture and Planning, Imam Abdulrahman Bin Faisal University, Dammam 31441, Saudi Arabia; 3 Department of Architecture, College of Architecture and Planning, Imam Abdulrahman Bin Faisal University, Dammam 32141, Saudi Arabia; 4 Department of Landscape Architecture, College of Architecture and Planning, Imam Abdulrahman Bin Faisal University, Dammam 31441, Saudi Arabia

**Keywords:** climate change, environmental pollution, health effects, landfilling, land degradation, solid waste management, storage and handling, recycling, risk exposure

## Abstract

Solid waste management (SWM) is one of the key responsibilities of city administrators and one of the effective proxies for good governance. Effective SWM mitigates adverse health and environmental impacts, conserves resources, and improves the livability of cities. However, unsustainable SWM practices, exacerbated by rapid urbanization and financial and institutional limitations, negatively impact public health and environmental sustainability. This review article assesses the human and environmental health impacts of SWM practices in the Global South cities that are the future of global urbanization. The study employs desktop research methodology based on in-depth analysis of secondary data and literature, including official documents and published articles. It finds that the commonplace SWM practices include mixing household and commercial garbage with hazardous waste during storage and handling. While waste storage is largely in old or poorly managed facilities such as storage containers, the transportation system is often deficient and informal. The disposal methods are predominantly via uncontrolled dumping, open-air incinerators, and landfills. The negative impacts of such practices include air and water pollution, land degradation, emissions of methane and hazardous leachate, and climate change. These impacts impose significant environmental and public health costs on residents with marginalized social groups mostly affected. The paper concludes with recommendations for mitigating the public and environmental health risks associated with the existing SWM practices in the Global South.

## 1. Introduction

Solid waste management (SWM) continues to dominate as a major societal and governance challenge, especially in urban areas overwhelmed by the high rate of population growth and garbage generation. The role of SWM in achieving sustainable development is emphasized in several international development agendas, charters, and visions. For example, sustainable SWM can help meet several United Nations’ Sustainable Development Goals (SDG), such as ensuring clean water and sanitation (SDG6), creating sustainable cities and inclusive communities (SDG11), mitigating climate change (SDG13), protecting life on land (SDG15), and demonstrating sustainable consumption and production patterns (SDG12) (https://sdgs.un.org/goals, accessed on 26 September 2022). It also fosters a circular urban economy that promotes reductions in the consumption of finite resources, materials reuse and recycling for waste elimination, pollution reduction, cost saving, and green growth

However, coupled with economic growth, improved lifestyle, and consumerism, cities across the globe will continue to face an overwhelming challenge of SWM as the world population is expected to rise to 8 billion by 2025 and to 9.3 billion by 2050, out of which around 70% will be living in urban areas [1,2]. In developing countries, most cities collect only 50–80% of generated waste after spending 20–50% of their budgets, of which 80–95% are spent on collecting and transporting waste [3,4]. Moreover, many low-income countries collect as low as 10% of the garbage generated in suburban areas, which contributes to public health and environmental risks, including higher incidents of diarrhea and acute respiratory infections among people, particularly children, living near garbage dumps [5]. Obstacles to effective municipal SWM include lack of awareness, technologies, finances, and good governance [6,7,8].

Removing garbage from homes and businesses without greater attention to what was then carried out with it has also been the priority of municipal SWM in several cities of developing countries [9]. In most developing countries, garbage collected from households is disposed of in landfills or dumpsites, the majority of which are projected to reach their capacities within a decade. The unsustainable approach of dumping or burning waste in an open space, usually near poor communities on the city edge, or throwing garbage into water bodies was an acceptable garbage disposal strategy. Similarly, several cities still use old-generation or poorly managed facilities and informal uncontrolled dumping or open-air waste burning. Often, these practices affect marginalized social groups near the disposal sites [10]. Moreover, this approach poses several sustainability problems, including resource depletion, environmental pollution, and public health problems, such as the spread of communicable diseases.

However, ever since the advent of the environmental movement in the 1960s, there has been a far-reaching appreciation of environmental and public health risks of unsustainable SWM practices. In the 1970s and onward, SWM was a technical issue to be resolved using technology; hence, the emphasis and investments were placed on garbage collection equipment [5]. Although modern technology can significantly reduce emissions of hazardous substances, by the 1990s, that viewpoint changed when municipalities become unable to evacuate and dispose of garbage effectively without the active involvement of service users and other stakeholders [5]. The inability of the public sector in the global South to deliver sufficient improvement of SWM, coupled with the pressure from the financial institutions and other donor agencies, led to privatization policies at the end of the decade. However, as privatization failed to provide municipal SWM services to the poor and marginalized communities, the current global thinking on addressing municipal SWM problems is changing. 

A more sustainable waste management approach prioritizes practices such as reduced production, waste classifications, reuse, recycling, and energy recovery over the common practices of landfilling, open dumps, and open incineration [11,12,13]. This approach, which is still at an early stage but getting increased attention in the Global South, is more inclusive and environment-friendly and has less negative impact on human health and the environment than the common practices [14,15,16]. As such, there is a need to assess SWM practices in the Global South and their impacts on environmental and human health because 90% of the expected growth in the urban population by 2050 is expected to happen here. So far, there are a few studies on the impacts of SWM practices on human health and the environment in the global regions.

Therefore, this review article addresses this knowledge gap by assessing the negative impacts of the dominant SWM practices on human and environmental health. Section 2 presents the research methodology. Section 3 reviews the major SWM practices in the Global South and assesses the environmental and public health implications of SWM practices in the Global South cities. While Section 4 discusses the implications of the findings and proffers recommendations that could help authorities to deal with SWM challenges and mitigate public and environmental health risks associated with unsustainable SWM practices, Section 5 concludes the paper.

## 2. Materials and Methods

**Figure 1:**
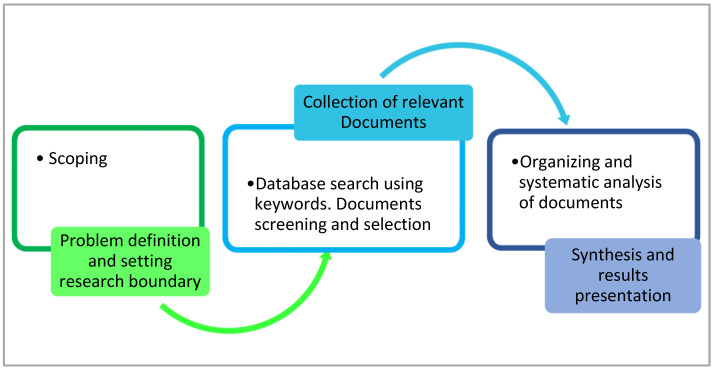
The flow chart of the research method (Source: [18] (p. 4)).

The present paper utilizes a desktop research method of collecting and analyzing relevant data from the existing literature, as utilized in some previous studies [17,18]. The method consists of three iterative stages shown in Figure 1: (a) scoping, (b) collecting relevant literature, and (c) data analysis. Firstly, the scoping stage involves defining and understanding the research problem under investigation and setting the study scope and boundary. The scope of the paper is to explore human and environmental impacts of SWM practices toward policy and practical recommendations for a more sustainable SWM system, with the Global South as the study boundary. This stage also helped identify relevant keywords to search for during the literature review in the second stage.

The second stage involved identifying and collecting relevant literature from online sources. The researchers utilized Google Scholar and Scopus databases to identify peer-reviewed academic works (peer-reviewed articles, conference proceedings, and books) as well as the gray literature. The literature that satisfied the following three inclusion criteria was identified and downloaded: (1) It is related to the study’s objective; (2) it is in the English language; and (3) it was published within the last twenty years, although some old documents about established concepts and approaches were also accessed. The downloaded gray literature includes newspaper articles, statistics, technical reports, and website contents from international development organizations such as the World Health Organization (WHO), the United Nations, and the World Bank.

In the last stage, the authors organized, analyzed, and synthesized the data collected from the literature. The downloaded works were organized according to the similarity of topics, even though some fit in more than one category. Then, each document was thoroughly examined, and themes concerned with SWM practices and their human and environmental impacts were collated, synthesized, and harmonized. Finally, the themes were summarized in Table A1, Table A2 and Table A3 (see Appendix A) and discussed. Implications and recommendations of the findings are then highlighted.

## 3. Results and Discussion

### 3.1. Solid Waste Management Practices in the Global South

Global municipal solid waste (MSW) generation rose from 1.3 billion tons in 2012 to 2.1 billion tons (0.74 kg/capita/day) as of 2016, which by 2050 is expected to increase by 70% to reach a total of 3.40 billion tons or 1.42 kg/capita/day [19]. The per capita MSW generation varies among regions and countries. In the EU (European Union), it ranges from 0.3–1.4 kg/capita/day [20], and in some African cities, the average is 0.78 kg/capita/day [21]. In Asia, urban areas generate about 760,000 tons of MSW per day, which is expected to increase to 1.8 million tons per day or 26% of the world’s total by 2025, despite the continent housing 53% of the world’s population [22,23]. In China, the total MSW generation was around 212 million tons (0.98 kg/capita/day) in 2006, out of which 91.4%, 6.4%, and 2.2% were disposed of via landfilling, incineration, and composting [24]. In 2010, only 660 Chinese cities produced about 190 million tons of MSW, accounting for 29% of the world’s total, while the total amount of solid waste in China could reach at least 480 million tons in 2030 [25]. In China, industrial waste (more than one billion tons) was five times the amount of MSW generated in 2002, which is expected to generate approximately twice as much MSW as the USA, while India will overtake the USA in MSW generation by 2030 [26]. 

In Malaysia, while the average rate of MSW generation was about 0.5–0.8 kg/person/day, Kuala Lumpur’s daily per capita generation rate was 1.62 kg in 2008 [27], which is expected to reach 2.23 kg in 2024 [28]. About 64% of Malaysia’s waste consists of household and office waste, 25% industrial waste, 8% commercial waste, and 3% construction waste [29]. In Sri Lanka, the assessed mean waste generation in 1999 was 6500 tons/day or 0.89 kg/cap/day, which is estimated to reach 1.0 kg/cap/day by 2025 [30]. With a 1.2% population growth rate, the total MSW generation in 2009 was approximately 7250 tons/day [31]. In Ghana, the solid waste generation rate was 0.47 kg/person/day, or about 12,710 tons per annum, consisting of biodegradable waste (0.318), non-biodegradable (0.096), and inert and miscellaneous waste (0.055) kg/person/day, respectively [32].

Moreover, global SWM costs are anticipated to increase to about $375.5 billion in 2025, with more than four-fold increases in lower- to middle-income countries and five-fold increases in low-income countries [33]. Globally, garbage collection, transportation, and disposal pose a major cost component in SWM systems [19]. Inadequate funding militates against the optimization of MSW disposal services. Table 1 compares the everyday SWM practices in low-, middle- and high-income countries according to major waste management steps. The literature indicates that waste generation rates and practices depend on the culture, socioeconomic status, population density, and level of commercial and industrial activities of a city or region.

### 3.2. Environmental and Public Health Impacts of SWM Practices in the Global South

(a)  *Weak and Inadequate SWM System*

Many problems in the cities of the global South are often associated with a weak or inadequate SWM system, which leads to severe direct and indirect environmental and public health issues at every stage of waste collection, handling, treatment, and disposal [30,31,32,33,34]. Inadequate and weak SWM results in indiscriminate dumping of waste on the streets, open spaces, and water bodies. Such practices were observed in, for example, Pakistan [35,36], India [37], Nepal [38], Peru [39], Guatemala [40], Brazil [41], Kenya [42], Rwanda [43], South Africa [44,45], Nigeria [46], Zimbabwe [47], etc.

The problems associated with such practices are GHG emissions [37,48], leachates [40,44,49], the spread of diseases such as malaria and dengue [36], odor [35,38,50,51], blocking of drains and sewers and subsequent flooding [52], suffocation of animals in plastic bags [52], and indiscriminate littering [38,39,53].

(b)  *Irregular Waste Collection and Handling*

Uncollected and untreated waste has socioeconomic and environmental costs extending beyond city boundaries. Environmental sustainability impacts of this practice include methane (CH_4_) emissions, foul odor, air pollution, land and water contamination, and the breeding of rodents, insects, and flies that transmit diseases to humans. Decomposition of biodegradable waste under anaerobic conditions contributes to about 18% and 2.9% of global methane and GHG emissions, respectively [54], with the global warming effect of about 25 times higher than carbon dioxide (CO_2_) emissions [55]. Methane also causes fires and explosions [56]. Emissions from SWM in developing countries are increasing due to rapid economic growth and improved living standards [57].

Irregular waste collection also contributes to marine pollution. In 2010, 192 coastal countries generated 275 million metric tons of plastic waste out of which up to 12.7 million metric tons (4.4%) entered ocean ecosystems [58]. Moreover, plastic waste collects and stagnates water, proving a mosquito breeding habitat and raising the risks of dengue, malaria, and West Nile fever [56]. In addition, uncollected waste creates serious safety, health, and environmental consequences such as promoting urban violence and supporting breeding and feeding grounds for flies, mosquitoes, rodents, dogs, and cats, which carry diseases to nearby homesteads [4,19,59,60].

In the global South, scavengers often throw the remaining unwanted garbage on the street. Waste collectors are rarely protected from direct contact and injury, thereby facing serious health threats. Because garbage trucks are often derelict and uncovered, exhaust fumes and dust stemming from waste collection and transportation contribute to environmental pollution and widespread health problems [61]. In India’s megacities, for example, irregular MSW management is one of the major problems affecting air and marine quality [62]. Thus, irregular waste collection and handling contribute to public health hazards and environmental degradation [63].

(c)  *Landfilling and Open Dumping*

Most municipal solid waste in the Global South goes into unsanitary landfills or open dumps. Even during the economic downturn during the COVID-19 pandemic, the amount of waste heading to landfill sites in Brazil, for example, increased due to lower recycling rates [64]. In Johor, Malaysia, landfilling destroys natural habitats and depletes the flora and fauna [65]. Moreover, landfilling with untreated, unsorted waste led to severe public health issues in South America [66]. Based on a study on 30 Brazilian cities, Urban and Nakada [64] report that 35% of medical waste was not properly treated before disposal, which poses a threat to public health, including the spread of COVID-19. Landfills and open dumps are also associated with high emissions of methane (CH_4_), a major GHG [67,68]. Landfills and wastewater release 17% of the global methane emission [25]. About 29 metric tons of methane are emitted annually from landfills globally, accounting for about 8% of estimated global emissions, with 1.3 metric tons released from landfills in Africa [7]. The rate of landfill gas production steadily rises while MSW accumulates in the landfill emissions. Released methane and ammonia gases can cause health hazards such as respiratory diseases [37,69,70,71]. Since methane is highly combustible, it can cause fire and explosion hazards [72].

Open dumping sites with organic waste create the environment for the breeding of disease-carrying vectors, including rodents, flies, and mosquitoes [40,45,51,73,74,75,76,77,78,79]. Associated vector-borne diseases include zika virus, dengue, and malaria fever [70,71,72,73,74,75,76,77,78,79,80]. In addition, there are risks of water-borne illnesses such as leptospirosis, intestinal worms, diarrhea, and hepatitis A [80,81].

Odors from landfill sites, and their physical appearance, affect the lives of nearby residents by threatening their health and undermining their livelihoods, lowering their property values [37,38,68,82,83,84]. Moreover, the emission of ammonia (NH_3_) from landfill sites can damage species’ composition and plant leaves [85]. In addition, the pollutants from landfill sites damage soil quality [73,84]. Landfill sites also generate dust and are sources of noise pollution [86].

Air and water pollution are intense in the hot and rainy seasons due to the emission of offensive odor, disease-carrying leachates, and runoff. Considerable amounts of methane and CO_2_ from landfill sites produce adverse health effects such as skin, eyes, nose, and respiratory diseases [69,87,88]. The emission of ammonia can lead to similar problems and even blindness [85,89]. Other toxic gaseous pollutants from landfill sites include Sulphur oxides [89]. While less than 20% of methane is recovered from landfills in China, Western nations recover up to 60% [90].

Several studies report leachate from landfill sites contaminating water sources used for drinking and other household applications, which pose significant risks to public health [36,43,53,72,75,83,91,92,93,94,95]. For example, Hong et al. [95] estimated that, in 2006, the amount of leachates escaping from landfill sites in Pudong (China) was 160–180 m^3^ per day. On the other hand, a properly engineered facility for waste disposal can protect public health, preserve important environmental resources, prevent clogging of drainages, and prevent the migration of leachates to contaminate ground and surface water, farmlands, animals, and air from which they enter the human body [61,96]. Moreover, heat in summer can speed up the rate of bacterial action on biodegradable organic material and produce a pungent odor [60,97,98]. In China, for example, leachates were not treated in 47% of landfills [99].

Co-mingled disposal of industrial and medical waste alongside municipal waste endangers people with chemical and radioactive hazards, Hepatitis B and C, tetanus, human immune deficiency, HIV infections, and other related diseases [59,60,100]. Moreover, indiscriminate disposal of solid waste can cause infectious diseases such as gastrointestinal, dermatological, respiratory, and genetic diseases, chest pains, diarrhea, cholera, psychological disorders, skin, eyes, and nose irritations, and allergies [10,36,60,61].

(d)  *Open Burning and Incineration*

Open burning of MSW is a main cause of smog and respiratory diseases, including nose, throat, chest infections and inflammation, breathing difficulty, anemia, low immunity, allergies, and asthma. Similar health effects were reported from Nepal [101], India [87], Mexico, [69], Pakistan [52,73,84], Indonesia [88], Liberia [50], and Chile [102]. In Mumbai, for example, open incineration emits about 22,000 tons of pollutants annually [56]. Mongkolchaiarunya [103] reported air pollution and odors from burning waste in Thailand. In addition, plastic waste incineration produces hydrochloric acid and dioxins in quantities that are detrimental to human health and may cause allergies, hemoglobin deficiency, and cancer [95,104]. In addition, smoke from open incineration and dumpsites is a significant contributor to air pollution even for persons staying far from dumpsites.

(e)  *Composting*

Composting is a biological method of waste disposal that entails the decomposing or breaking down of organic wastes into simpler forms by naturally occurring microorganisms, such as bacteria and fungi. However, despite its advantage of reducing organic waste by at least half and using compost in agriculture, the composting method has much higher CO_2_ emissions than other disposal approaches. In Korea, for example, composting has the highest environmental impact than incineration and anaerobic digestion methods [105]. The authors found that the environmental impact of composting was found to be 2.4 times higher than that of incineration [105]. Some reviews linked composting with several health issues, including congested nose, sore throat and dry cough, bronchial asthma, allergic rhinitis, and extrinsic allergic alveolitis [36,106].

## 4. Implications and Recommendations

As discussed in the section above, there are many negative impacts of unsustainable SWM practices on the people and the environment. Although all waste treatment methods have their respective negative impacts, some have fewer debilitating impacts on people and the environment than others. The following is the summary of key implications of such unsustainable SWM practices.

- Uncollected organic waste from bins, containers and open dumps harbors rodents, insects, and reptiles that transmit diseases to humans. It also produces odor due to the decomposition of organic wastes, especially in the summer, and leachates that migrate and contaminate receiving underground and surface waters.
- Open dumps and non-engineered landfills release methane from decomposing biodegradable waste under anaerobiotic conditions. Methane is a key contributor to global warming, and it can cause fires and explosions.
- Non-biodegradable waste, such as discarded tires, plastics, bottles, and tins, pollutes the ground and collects water, thus creating breeding grounds for mosquitoes and increasing the risk of diseases such as malaria, dengue, and West Nile fever.
- Open burning of MSW emits pollutants into the atmosphere thereby increasing the incidences of nose and throat infections and inflammation, inhalation difficulties, bacterial infections, anemia, reduced immunity, allergies, and asthma.
- Uncontrolled incineration causes smog and releases fine particles, which are a major cause of respiratory disease. It also contributes to urban air pollution and GHG emissions significantly.
- Incineration and landfilling are associated with reproductive defects in women, developmental defects in children, cancer, hepatitis C, psychosocial impacts, poisoning, biomarkers, injuries, and mortality.

Therefore, measures toward more sustainable SWM that can mitigate such impacts must be worked out and followed. The growing complexity, costs, and coordination of SWM require multi-stakeholder involvement at each process stage [7]. Earmarking resources, providing technical assistance, good governance, and collaboration, and protecting environmental and human health are SWM critical success factors [47,79]. As such, local governments, the private sector, donor agencies, non-governmental organizations (NGOs), the residents, and informal garbage collectors and scavengers have their respective roles to play collaboratively in effective and sustainable SWM [40,103,107,108]. The following are key practical recommendations for mitigating the negative impacts of unsustainable SWM practices enumerated above.

First, cities should plan and implement an integrated SWM approach that emphasizes improving the operation of municipalities to manage all stages of SWM sustainably: generation, separation, transportation, transfer/sorting, treatment, and disposal [36,46,71,77,86]. The success of this approach requires the involvement of all stakeholders listed above [109] while recognizing the environmental, financial, legal, institutional, and technical aspects appropriate to each local setting [77,86]. Life Cycle Assessment (LCA) can likewise aid in selecting the method and preparing the waste management plan [88,110]. Thus, the SWM approach should be carefully selected to spare residents from negative health and environmental impacts [36,39,83,98,111]. 

Second, local governments should strictly enforce environmental regulations and better monitor civic responsibilities for sustainable waste storage, collection, and disposal, as well as health hazards of poor SWM, reflected in garbage littering observable throughout most cities of the Global South [64,84]. In addition, violations of waste regulations should be punished to discourage unsustainable behaviors [112]. Moreover, local governments must ensure that waste collection services have adequate geographical coverage, including poor and minority communities [113]. Local governments should also devise better SWM policies focusing on waste reduction, reuse, and recycling to achieve a circular economy and sustainable development [114,115]. 

Third, effective SWM requires promoting positive public attitudes toward sustainable waste management [97,116,117,118]. Therefore, public awareness campaigns through print, electronic, and social media are required to encourage people to desist from littering and follow proper waste dropping and sorting practices [36,64,77,79,80,82,91,92,119]. There is also the need for a particular focus on providing sorting bins and public awareness about waste sorting at the source, which can streamline and optimize subsequent SWM processes and mitigate their negative impacts [35,45,46,64,69,89,93]. Similarly, non-governmental and community-based organizations can help promote waste reduction, separation, and sorting at the source, and material reuse/recycling [103,120,121,122]. In Vietnam, for example, Tsai et al. [123] found that coordination among stakeholders and appropriate legal and policy frameworks are crucial in achieving sustainable SWM.

Fourth, there is the need to use environmentally friendly technologies or upgrade existing facilities. Some researchers prefer incineration over other methods, particularly for non-recyclable waste [44,65]. For example, Xin et al. [124] found that incineration, recycling, and composting resulted in a 70.82% reduction in GHG emissions from solid waste in Beijing. In Tehran city, Iran, Maghmoumi et al. [125] revealed that the best scenario for reducing GHG emissions is incinerating 50% of the waste, landfilling 30%, and recycling 20%. For organic waste, several studies indicate a preference for composting [45,51,75] and biogas generation [15,42,68]. Although some researchers have advocated a complete ban on landfilling [13,42], it should be controlled with improved techniques for leak detection and leachate and biogas collection [126,127]. Many researchers also suggested an integrated biological and mechanical treatment (BMT) of solid waste [66,74,95,119]. In Kenya, the waste-to-biogas scheme and ban on landfill and open burning initiatives are estimated to reduce the emissions of over 1.1 million tons of GHG and PM2.5 emissions from the waste by more than 30% by 2035 [42]. An appropriately designed waste disposal facility helps protect vital environmental resources, including flora, fauna, surface and underground water, air, and soil [128,129].

Fifth, extraction and reuse of materials, energy, and nutrients are essential to effective SWM, which provides livelihoods for many people, improves their health, and protects the environment [130,131,132,133,134,135,136]. For example, recycling 24% of MSW in Thailand lessened negative health, social, environmental, and economic impacts from landfill sites [89]. Waste pickers play a key role in waste circularity and should be integrated into the SWM system [65,89,101,137], even to the extent of taking part in decision-making [138]. In addition, workers involved in waste collection should be better trained and equipped to handle hazardous waste [87,128]. Moreover, green consumption, using bioplastics, can help reduce the negative impacts of solid waste on the environment [139]. 

Lastly, for effective SWM, local authorities should comprehensively address SWM challenges, such as lack of strategic SWM plans, inefficient waste collection/segregation and recycling, insufficient budgets, shortage of qualified waste management professionals, and weak governance, and then form a financial regulatory framework in an integrated manner [140,141,142]. Effective SWM system also depends on other factors such as the waste generation rate, population density, economic status, level of commercial activity, culture, and city/region [37,143]. A sustainable SWM strives to protect public health and the environment [144,145].

## 5. Conclusions

As global solid waste generation rates increase faster than urbanization, coupled with inadequate SWM systems, local governments and urban residents often resort to unsustainable SWM practices. These practices include mixing household and commercial garbage with hazardous waste during storage and handling, storing garbage in old or poorly managed facilities, deficient transportation practices, open-air incinerators, informal/uncontrolled dumping, and non-engineered landfills. The implications of such practices include air and water pollution, land degradation, climate change, and methane and hazardous leachate emissions. In addition, these impacts impose significant environmental and public health costs on residents with marginalized social groups affected mostly.

Inadequate SWM is associated with poor public health, and it is one of the major problems affecting environmental quality and cities’ sustainable development. Effective community involvement in the SWM requires promoting positive public attitudes. Public awareness campaigns through print, electronic, and social media are required to encourage people to desist from littering and follow proper waste-dropping practices. Improper SWM also resulted in water pollution and unhealthy air in cities. Future research is needed to investigate how the peculiarity of each Global South country can influence selecting the SWM approach, elements, aspects, technology, and legal/institutional frameworks appropriate to each locality.

## Figures and Tables

**Table 1:** Common MSW management practices by country’s level of economic development (adapted from [34]).

Activity	Low-Income Countries	Middle-Income Countries	High-Income Countries
Source Reduction	Low per capita waste generation rates, no organized SWM program, high reuse rate.	Some source reduction elements but rarely incorporated into an organized SWM program.	SWM programs emphasize the three “Rs”: reduce, reuse, and recycle. More producer responsibility.
Collection	Infrequent and inefficient. Serves mainly high visibility areas, the wealthy, and businesses willing to pay. A high fraction of inert and compostable waste impact collection. The overall collection is less than 50%.	Improved collection and transportation in residential areas. Large vehicle fleet and mechanization. The overall collection rate is from 50% to 80%. Transfer stations are gradually incorporated into the SWM system.	More than 90% collection rate. Compactor and well-mechanized trucks, and transfer stations are common. Waste volume is a major consideration. Aging collection workers are often considered in system design.
Recycling	Informal sector recycling by scavengers is dominant. High recycling rates for local and international markets. Imports of materials for recycling, including hazardous goods such as e-waste and shipbreaking. Recycling markets are unregulated and include several “middlemen”. Large price fluctuations.	Informal recycling, high technology sorting, and processing facilities. Relatively high recycling rates. Materials are often imported for recycling. Recycling markets are mostly regulated. Material prices fluctuate considerably.	Recyclable material collection, high-technology sorting, and processing facilities are common and regulated. Increased attention towards long-term markets. Overall, recycling rates are higher than in middle- and low-income countries. Informal recycling still exists (e.g., collecting aluminum cans). Extended product responsibility is common.
Composting	It is rarely performed formally, albeit the waste consists of a high percentage of organic material. Markets for, and awareness of, compost are lacking.	It is not widespread. Largescale composting facilities are mostly unsuccessful because of contamination and operating costs (little waste separation); some small-scale composting projects at the community/neighborhood level are more sustainable than the large-scale. Growing use of anaerobic digestion.	It is widespread in backyard and large-scale facilities. The waste consists of smaller portions of organic matter than low- and middle-income countries. More source segregation makes composting easier. Anaerobic digestion is gaining popularity. Odor control is critical.
Incineration	It is uncommon and mostly unsuccessful due to high capital, technical, and operation costs, the high moisture content in the waste, and the high proportion of inert waste.	A few incinerators operate but experience financial and operational difficulties. Air pollution control equipment is not advanced and is often bypassed. Lack of emissions monitoring. Facilities are often driven by subsidies as construction and operation costs are prohibitive.	Predominant in areas where land is scarce or expensive (e.g., islands). It is mostly subjected to environmental control to regulate and monitor emissions. It recovers energy but it is about at least three-folds the cost of landfilling per ton.
Landfilling and open dumping	Open dumping of waste and low-technology landfill sites. High pollution to nearby aquifers, water bodies, and communities. Regularly receive medical waste. Waste is often burned. Significant health impacts on workers and residents.	Sanitary landfills with some environmental controls often exist. Open dumping of garbage is widespread. Projects for landfill gas collection under clean development mechanism are commonplace.	Sanitary landfills combined with liners, leak detection, and leachate collection systems. Gas collection and treatment systems. It is often problematic to open new landfills due to concerns of neighboring residents. Post-closure use of sites is increasingly important, e.g., golf courses and parks.
Costs	Waste collection costs represent 80–90% of the municipal SWM budget. Local governments regulate waste fees, but the fee collection system is inefficient. Only a small proportion of the budget is allocated toward disposal.	Collection costs represent 50% to 80% of the municipal SWM budget. Some local and national governments regulate waste fees and more innovation in fee collection, e.g., included in electricity or water bills. More mechanized collection fleets and disposal expenditures are higher than in low-income countries.	Collection costs can represent less than 10% of the budget. Large budget allocations to intermediate waste treatment facilities. Upfront community participation reduces costs and increases options available to waste planners (e.g., recycling and composting).

## Data Availability

No data were reported in this review article.

## Appendix A

**Table A1:** Reviewed literature on the impacts of SWM practices in Asia (compiled by authors).

Author	Study Area	Study Aim	Impacts on Humans	Impacts on the Environment	Recommendations/Implications
Akmal & Jamil [36]	Rawalpindi and Islamabad, Pakistan	Examines the relationship between residents’ health and dumpsite exposure.	▪ Open dumpsites and haphazard waste disposal lead to malaria and dengue fever. ▪ Health risk due to water contamination from dumpsites. ▪ Respiratory diseases, including asthma, skin diseases, and diarrhea due to proximity to dumpsites.	▪ Groundwater contamination from leachate from landfill sites ▪ Land pollution due to the emptying of waste in drains, open sewers, roads, streets, and railway tracts.	▪ Locating landfill sites in the suburbs and removing illegal dumpsites within the residential areas. ▪ Public awareness campaigns on the adverse effects of living around dump sites.
Hong et al. [95]	Pudong, China	Assesses the environmental impacts of five SW treatment options	▪ Incinerating plastic wastes produces HCl acid and dioxins, which are detrimental to human health	▪ Leachates from landfills and open incineration sites contaminate soil, surface water, and groundwater ▪ Global warming due to CO_2_ and acidification from NOx and SO_2_	▪ Integrate BMT into the SWM system to reduce emissions and maximize recycling
Gunamantha [88]	Kartamantul region, Yogyakarta, Indonesia	Compares five energetic valorization alternative scenarios and existing SW treatment.	▪ CH_4_ and CO_2_ emissions from landfill sites produce adverse health effects such as skin, eyes, nose, and respiratory diseases.	▪ Emissions of CH_4_ and CO_2_ gases from landfill sites aggravated global warming challenges.	▪ Use the LCA approach to assist in decision-making on the SWM plan
Abba et al. [65]	Johor Bahru, Malaysia	Assesses stakeholder opinion on the existing and future environmental impacts of household solid waste disposal.	▪ Lung and eye inflammation problems due to air pollution	▪ Emissions of CO_2_, N_2_O, and NH_3_ increase climate change challenges. ▪ Leachates contaminate water bodies ▪ Depletion of fauna and flora due to landfills.	▪ Incineration protects stream ecology, fauna, flora, and air quality, enhances environmental visibility, and optimizes land use.
Fang et al. (2012) [85]	Shanghai, China	Identifies different sources of MSW odor compounds generated by landfill sites.	▪ Emissions of NH_4_ cause harm to the respiratory tract, eyes, nose, lungs, etc.	▪ Emissions of NH_3_ damage species composition, plant leaves, etc.	▪ Provide engineered landfills
Menikpura et al. [89]	Nonthaburi municipality, Bangkok, Thailand	Explores recycling activities’ effects on the sustainability of SWM practices.	▪ Emission of hazardous gasses from landfill sites such as CH_4_, NH_3_, and NOx are associated with human toxicity and ailments.	▪ Significant damage to the ecosystem due to acidifying and eutrophying substances emissions.	▪ Promote more recycling of MSW.
Mongkolnchaiarunya [103]	Yala Manucipality, Thailand	Investigates the possibilities of integrating alternative SW solutions with local practices.	▪ Open burning of waste causes respiratory ailments and odors	▪ Air and soil pollution due to waste burning. ▪ Negatively affect the aesthetic landscape of the environment.	▪ Partnerships between sectors to inculcate new ideas, information, and skills in solid-waste management issues.
De & Debnath [98]	Kolkata, India	Investigates the health effects of solid waste disposal practices.	▪ Open dumping has caused associated health risks, including malaria, dengue, and diarrhea.	▪ Water and air pollution are due to indiscriminate waste disposal on streets, drains, open spaces, and water bodies.	▪ Proper MSW dumping sites to reduce land degradation and human health impacts
Suthar & Sajwan [83]	Dehradun city, India	Proposes a new solid waste disposal site	▪ Odor problems among residents living close to the landfill sites or nearby locations.	▪ Leachates pollute surface and groundwater.	▪ Physical and chemical components are the key factor in site selection
Phillips & Mondal [68]	Varanasi, India	Evaluates the sustainability of solid waste disposal options	▪ Visual and odor impacts due to open dumping. ▪ Human health hazards due to CH_4_ and CO_2_	▪ Leachate causes hazards to the environment, surface, and groundwater bodies.	▪ Gasification was the most effective and sustainable solid waste disposal option.
Ramachandra et al. [37]	Bangalore, India	Assesses the composition of waste for its management and treatment	▪ Indiscriminate disposal of waste has caused visual impacts on the environment. ▪ Emissions of CO_2_ and CH_4_ cause likely adverse health effects.	▪ GHG emissions due to indiscriminate disposal of waste contribute to global warming ▪ Water and land pollution.	▪ Integrated SWM strategy to handle the organic components through policy interventions and technology.
Pokhrel & Viraraghavan [38]	Kathmandu Valley, Nepal	Evaluates SWM practices in Nepal.	▪ Haphazard disposal of solid wastes affects the residents’ lives due to odor and associated health effects.	▪ Polluted riverbanks and water resources ▪ The tourism industry is severely affected by the open dumping of solid wastes.	▪ Composting. ▪ Ban indiscriminate disposal of solid wastes
Dangi et al. [93]	Tulsipur, Nepal	Investigates household SWM options.	▪ Contaminated water consumption by citizens results in several health effects.	▪ Water and soil contamination due to the absence of a leachate treatment facility	▪ Recycling and composting.
Islam (2016) [82]	Dhaka, Bangladesh	Develops an effective SWM and recycling process for Dhaka city	▪ Emission from open-air dumping practices causes health threats to residents. ▪ Nuisance and aesthetic issues due to strong odor.	▪ Pollution of water bodies. ▪ CO_2_ and CH_4_ emissions pollute the environment.	▪ Strict rules on haphazard solid waste disposal and public awareness campaigns.
Das et al. [101]	Kathmandu valley, Nepal	Estimates the amount of MSW burnt in five municipalities.	▪ Open-air burning causes health-threatening effects, such as respiratory infections, allergic hypersensitivity, and heart diseases.	▪ Global warming problem due to CO_2_ and CH_4_ emissions	▪ Improve waste segregation at the source and waste collection points. ▪ Penalty for open burning and indiscriminate waste disposal
Usman et al. [84]	Faisalabad, Pakistan	Investigates the impacts of open dumping on groundwater quality	▪ Unpleasant odors, visual impacts, and risks to residents’ health ▪ CO_2_ and CH_4_ emissions from open-air burning.	▪ Soil quality degradation by pollutants ▪ Surface and ground water contamination by leachates’ percolation.	▪ Effective monitoring and supervision for waste disposal and leachate management.
Nisar et al. (2008) [73]	Bahawalpur City, Pakistan	Explores the sources and impacts of SWM practices	▪ Breeding of disease-carrying vectors, including rodents, mosquitoes, etc. ▪ Severe infections due to air and water pollutants.	▪ Land degradation ▪ Decrease in land values ▪ Air and water pollution	▪ The community’s health and environmental problems are due to poor SWM practices.
Ejaz et al. (2010) [52]	Rawalpindi city, Pakistan	Identifies the causes of illegal dumping of SWM.	▪ Unhygienic conditions for residents due to odor, leachates, and associated emissions. ▪ Spread of infections due to breeding of diseases-carrying vectors.	▪ Blocking of drains and sewer system triggering periodic flooding. ▪ Littering of polythene bags causes aesthetic nuisance and death in animals. ▪ Air and water pollution	▪ Public awareness, community participation ▪ Provide resources, equipment, and funding
Batool & Chaudhry [35]	Lahore, Pakistan	Evaluates the effect of MSW management practices on GHG emissions.	▪ Odor due to indiscriminate disposal of waste causes a threat to human health. ▪ CO_2_ and CH_4_ emissions are causing associated health risks.	▪ Wastes are disposed of on vacant land, excavations, flood plains, and water bodies. ▪ Land degradation and soil deterioration. ▪ Air pollution from CO_2_ and CH_4_ emissions.	▪ Recycling to reduce the purchase of expensive lands for landfills. ▪ Bio-gasification.
Hoang & Fogarassy [74]	Hanoi, Vietnam	Explores the most sustainable MSW management options using MCDA.	▪ Threats to public health due to GHG emission and water contamination. ▪ Pungent odor, which is a detriment to health.	▪ Air and water pollution due to GHG emissions and leachate ▪ Land and soil deterioration. ▪ Visual impacts due to overcrowded landfills.	▪ Mechanical–biological treatment (MBT) plants as the sustainable solution for MSW systems.
Ansari [86]	Bahrain	Proposes an integrated and all-inclusive SWM system	▪ Public health risks due to leachates and landfill gas generation. ▪ Fire hazard. ▪ Odor generation.	▪ Landscape and soil quality destruction. ▪ Dust generation. ▪ Groundwater contamination. ▪ Noise and air pollution	▪ A partnership between government and stakeholders to achieve a sustainable integrated SWM system.
Clarke et al. [53]	Qatar	To collect data about residents’ specific opinions concerning SW strategies.	▪ Emissions from landfill sites are associated with organic wastes. ▪ Leachates contaminate ground and surface water bodies.	▪ Garbage disposal on beaches. ▪ Indiscriminate littering of plastic and paper wastes. ▪ Unhealthy waste food disposal.	▪ Behavioral change among residents to achieve transformational and sustainable SWM.
Ossama et al. [115]	Saudi Arabia	Reviews municipal SWM practices in Saudi Arabia	▪ Generating landfill gases such as CH_4_ causes infection in humans. ▪ Leachates cause harm to humans.	▪ Water bodies polluted by leachates from landfill sites ▪ Air pollution from the disposal sites	▪ Recycling, natural resources conservation, and reducing pollution and landfilling.
Brahimi et al. [104]	India	Explores the potential of waste-to-energy in India	▪ Infections from contaminated ground and surface water bodies. ▪ Producing cancer-forming chemicals such as dioxin and furans due to incineration ▪ Respiratory infections from incineration and landfilling. ▪ Bacterial infection, hemoglobin deficiency, and allergy due to poor SWM.	▪ Water, soil, noise, and air pollution from landfilling and incineration. ▪ Global warming from open-air burning and hazardous gases emissions from landfilling and incineration. ▪ Odor nuisance.	▪ Enact policies to improve and encourage WTE industry and support the investors.

**Table A2:** Reviewed literature on the impacts of SWM practices in South America (compiled by authors).

Author	Study Area	Aim	Impacts on Humans	Impacts on the Environment	Recommendations/Implications
McAllister [39]	Peru, South America	To conduct a comprehensive review on the impact of inadequate SWM practices on natural and human environments	▪ Spread of diseases. ▪ Threats to public health.	▪ The occurrence of littering. ▪ Unsanitary urban conditions.	▪ Public awareness, attitude change, and waste prevention campaigns. ▪ Educate the citizenry on waste reduction and separation as a national policy and waste-minimization enactment.
Bezama et al. [66]	Concepción (Chile) province and the city of Estrela (Brazil)	To analyze the suitability of mechanical biological treatment of municipal solid waste in South America.	▪ Landfilling of unsorted and untreated waste causes threats to public health.	▪ Environmental pollution due to unsorted and untreated waste	▪ Mechanical biological treatment (MBT) of wastes before landfilling could be suitable for municipal SWM in South American countries.
Ansari [120]	Guyana (South America)	To develop effective and low-cost technologies for organic waste recycling	▪ Odor nuisance and bacterial infections, including lungs, nose, sinus, and throat infections ▪ Leachate polluting water bodies cause stomach infections.	▪ Water pollution. ▪ Air pollution. ▪ Environment deterioration.	▪ Combine effective technologies to enhance agricultural enrichment in developing countries.
Hoornweg & Giannelli [25]	Latin America and the Caribbean	To integrate the private sector to harness incentives in managing MS.W. in Latin America and the Caribbean.	▪ CH_4_ gas released from landfills is detrimental to public health.	▪ Air pollution due to CH_4_ emissions from landfills	▪ Private participation. ▪ Small-scale providers. ▪ Integrate waste pickers into the SWM system. ▪ Upgrading landfills. ▪ Policies and incentives as key tools for an effective SWM system. ▪ Build municipal capacity. ▪ Tap from carbon finance.
Olay-Romero et al. [78]	Sixty-six Mexican municipalities, Mexico	To propose a basic set of indicators to analyze technical aspects of street cleaning, collection, and disposal.	▪ Open-dumping practices produce disease vectors. ▪ Landfills and open dump sites generate hazardous gases.	▪ Open dumps and landfill sites pollute water, air, and land.	▪ Increase the coverage of the collection services. ▪ Improve the conditions of the disposal sites. ▪ The proposed indicators can systematize the supervision and detection of areas of improvement in the MSWM.
Urban & Nakada [64]	Thirty Brazilian cities	Assess environmental impacts caused by shifts in solid waste production and management due to the COVID-19 pandemic.	▪ Improper disposal of facemasks may increase the spread of COVID-19. ▪ Economic and environmental losses due to sales of recyclable materials during the suspension of recycling programs and reducing landfill lifespan	▪ Hindrance to natural resources for not being saved due to recycling programs’ suspension	▪ Increase recycling capacity and environmental education, for example, using disposable packages and utensils from online shopping and food delivery. ▪ Encourage waste pickers’ training. ▪ Monitor the installed capacity and production for medical waste treatment. ▪ Limit using disposable masks to health personnel only and reusable fabric facemasks to the general population.
Gavilanes-Terán et al. [75]	Ecuadorian province of Chimborazo, Ecuador.	Categorize organic wastes from the agroindustry and evaluate their potential use as soil amendments.	▪ Disease transmissions by vectors formed due to indiscriminate organic waste disposal. ▪ Leachates cause detrimental impacts on human health.	▪ Odor generation ▪ Water and air contaminations	▪ The use of conditioning treatments, such as composting, is essential before using the residues for agricultural uses. ▪ The wastes must be fully categorized before using for agricultural purposes.
Pérez et al. [102]	City of Valdivia (Chile)	Holistic environmental assessment perspective for municipal SWM.	▪ Respiratory diseases triggered by GHGs’ emissions	▪ Environmental pollution such as air pollution.	▪ Using Life Cycle Assessment (LCA) approach allows the assessment of the potential impact of MSW management and disposal technologies.
Yousif & Scott [40]	Mazatenango, Guatemala	Examines the problems of SWM concerning administration, collection, handling, and disposal	▪ Spread of infection from disease-carrying vectors such as rats and flies. ▪ Skin and respiratory infections and physical disabilities from direct contact with waste.	▪ Odor generation from indiscriminate dumping and proliferation of refuse on streets ▪ Environmental pollution from leachates and emission of landfill gases	▪ Strengthen the relationships among the stakeholders involved in the administrative, economic, social, and environmental aspects of SWM.
Azevedo et al. [70]	Rocinha, Brazil	To develop a SWM framework from the sustainable supply chain management (SSCM) perspective.	▪ Transmission of diseases such as dengue and leptospirosis. ▪ Respiratory infection due to hazardous gases emitted from the disposal sites.	▪ Contaminated ditches. ▪ Uneven and indiscriminate dumps. ▪ Air and water pollution. Debris flows into rivers and the ocean.	▪ Solve basic social issues related to security, education, and infrastructure. ▪ Proposed an SSCM framework and strategies for better SWM
Penteado & de Castro [80]	Brazil	Reviews the main SWM recommendations during the pandemic.	▪ Public exposure to waterborne infections such as intestinal worms, diarrhea, dengue fever, hepatitis A, leptospirosis, and Zika virus	▪ Water, air, and land pollution	▪ Public awareness and engagement campaigns to reduce infectious waste disposals.
Pereira & Fernandino [77]	Mata de São João, Brazil	Evaluates waste management quality and tests the applicability of a system of indicators	▪ The proliferation of disease-carrying vectors. ▪ Waterborne and airborne diseases.	▪ Landscape and public space pollution.	▪ Establish an integrated MSWM plan. ▪ Provide a selective waste collection plan. ▪ Environmental education programs. ▪ Establish social inclusion program for the municipality’s recyclable material collectors.
Buenrostro & Bocco [121]	Mexico	Explores the causes and implications of MSW generation patterns	▪ Public health threats due to lack of sanitary landfills	▪ Unplanned sanitation landfills pollute the environment	▪ Provide financial, technical, and human resources. ▪ Involve skilled personnel in the decision-making process.
Juárez-Hernández [119]	Mexico City, Mexico	Evaluates MSW practices in the megacity.	▪ Poorly managed MSW causes health and social issues for the residents.	▪ Environmental pollution due to poor municipal SWM.	▪ Mechanical–biological pre-treatment, composting, refuse-derived fuel production, and material recovery facilities to achieve sustainable MSWM.
de Morais Lima & Paulo [41]	Quilombola communities, Brazil	Proposes a new approach for SWM using risk analysis and complementary sustainability criteria	▪ Indiscriminate disposal of wastes threatens public health. ▪ Disease transmissions by vectors.	▪ Air pollution due to open burning of dry waste. ▪ Garbage disposal on land and water bodies.	▪ Recommend a combination of household composting and source separation of dry waste.
Coelho & Lange [76]	Rio de Janeiro, Brazil.	Investigates sustainable SWM solutions	▪ Poor SWM threatens public health due to the generation of disease vectors.	▪ Environmental degradation such as water and air pollution	▪ New strategies that are more environmentally friendly and sustainable should be implemented.
Aldana-Espitia et al. [69]	City of Celaya, Guanajuato, Mexico.	Analyzes the existing municipal SWM process	▪ The emission of hazardous gases is detrimental to human health. ▪ Odor and respiratory infections. ▪ Waterborne diseases due to leachates.	▪ Air and water pollution. ▪ Land contamination due to indiscriminate dumping.	▪ Capture and use landfill gases for power generation. ▪ Recover and recycle materials to mitigate environmental impacts.
Silva & Morais [81]	Craft brewery, the northeastern Brazilian city	Develops a collaborative approach to SWM.	▪ Air- and waterborne-related diseases.	▪ Land, water, and air pollution.	▪ Sustainable responsibilities for the strategic performance of SWM in transitioning to a circular economy.
Morero et al. [71]	Cities in Argentina	Proposes a mathematical model for optimal selection of municipal SWM alternatives	▪ Public health threats from informal landfills	▪ High environmental pollution from the landfills	▪ Waste sorting and recycling can increase profitability in small populations.
Bräutigam et al. [72]	Metropolitan Region of Santiago de Chile	Identifies the technical options for SWM to improve the sustainability of the system.	▪ Emissions and odor from landfills cause fire risks and harm to human health ▪ Leachate contaminates water bodies and causes waterborne infections.	▪ Water and air pollutions due to leachate and landfill gas emissions.	▪ Segregated collection of biowaste for diverting MSW from landfills and reducing associated negative impacts.
Vazquez et al. [110]	Bahia Blanca, Argentina.	Assesses the type and amount of MSW generated in the city	▪ Hazardous gases emitted from open-air dumps affect human health.	▪ Air and water pollution due to open-air dumps	▪ Appropriate size and location of disposal facilities for source separation and a redesign of MSW collection routes. ▪ Recycle components of MSW to create new jobs ▪ Improve the working conditions of workers.
Zarate et al. [91]	San Mateo Ixtatán, Guatemala	Implements SWM program to address one of the public health needs	▪ Low-quality drinking water due to pollution could cause waterborne diseases in humans.	▪ Water contamination due to indiscriminate dumping of garbage.	▪ Educate students and the community on the key SWM principles.
Rodic-Wiersma & Bethancourt [107]	Guatemala City, Guatemala	Evaluates the present situation of the SWM system	▪ Adverse effects of dumpsites such as air pollution, leachates, and the proliferation of disease-carrying vectors. ▪ Water contaminations could cause waterborne diseases.	▪ Water pollution due to dumping of SW and discharging of sewage into rivers. ▪ SW reduction through recycling programs	▪ The informal sector is essential in recycling, which helps alleviate poverty, reduce the importation of materials, and conserve resources. ▪ Public participation and consultation are essential for the SWM and a cleaner living environment.
Burneo et al. [113]	Cuenca (Ecuador)	Evaluates the role of waste pickers and the conditions of their activities	▪ Threat to public health via increased GHG emissions	▪ Reduction in GHG emissions by using recycled urban waste	▪ Public campaigns to collect recyclers together and encourage public participation. ▪ More economic investment to deploy new technologies to optimize waste collection and processing systems.

**Table A3:** Reviewed literature on the impacts of SWM practices in Africa (compiled by authors).

Author	Study Area	Study Aim	Impacts on Humans	Environment Impacts	Recommendations/Implications
Dianati et al. [42]	Kisumu, Kenya	Explores the impact on PM_2.5_ and GHG emissions of the waste-to-biogas scheme	▪ Adverse health risks from GHG emissions and atmospheric pollutants originating from open burning	▪ Indiscriminate waste disposal and burning cause environmental challenges, including air and water pollution.	▪ Impose a regulatory ban on landfilling and open burning.
Kabera et al. [143]	Kigali, Rwanda, and Major cities of East Africa	Benchmarks and compares the performance of SWM and recycling systems	▪ Threat to public health from open dumps and burning of waste	▪ Water and air pollution from uncontrolled dumping and open burning	▪ Eliminate uncontrolled dumping and open-air burning of waste ▪ Improve recycling, segregate waste at source, separate collection, and learn from experience
Kadama [43]	The North West Province of South Africa	Formulates a new approach to SWM based on the business process re-engineering principle.	▪ Pollution causes water and airborne diseases and generates disease-carrying vectors.	▪ Leachates contaminate surface and groundwater bodies.	▪ A regional logistical framework that offers a sustainable, cost-effective, incorporated waste management system
Owojori et al. [45]	Limpopo Province, South Africa	Determines the differences among waste components.	▪ Organic wastes attract disease-carrying vectors.	▪ Environment deterioration due to indiscriminate waste disposal	▪ Recycling to reduce the amount of waste transported to landfills.
Ayeleru et al. [116]	Soweto, South Africa	Evaluates the cost-benefit analysis of setting up a recycling facility.		▪ Environmental pollution due to poor SWM practices	▪ Recycling is essential and has potential for job generation.
Friedrich & Trois [48]	eThekwiniMunicipality, South Africa	Estimates the current and future GHG emissions from garbage.	▪ Respiratory illness from emitting GHG.	▪ Air pollution and odor nuisance.	▪ Life cycle-based system can support municipal SWM decision-making, planning, and development of future SWM strategies
Nahmana & Godfreyb [92]	South Africa	Explores the opportunities and constraints to implementing economic instruments for SWM	▪ Air and waterborne infections due to illegal dumping and inadequate waste collection services	▪ Air pollutants due to GHG emissions and leachates contaminating water bodies	▪ Cost recovery by promulgating SWM bill ▪ Education and awareness, political involvement ▪ Capacity and infrastructure development in SWM practices ▪ Enforcement of existing instruments
Filimonau & Tochukwu [114]	Lagos, Nigeria	Explores SWM practices in selected hotels in Lagos.	▪ Health challenges due to insufficient environmental awareness and staff disengagement.	▪ Environmental degradation due to low-quality SWM infrastructure	▪ Raising environmental awareness among employees and guests. ▪ Government participation is essential in environmental awareness campaigns for hoteliers and guests.
Trois & Vaughan-Jones [118]	Africa	Proposes a plan for sustainable SWM	▪ GHG emissions from waste aggravate the effects of climate change which has adverse impacts on human health and the economy	▪ Environmental pollution due to GHG emissions from landfills. ▪ Land and water pollution	▪ Low-cost mechanical-biological pre-treatment and composting for sustainable SWM
Parrot & Dia [51]	Yaoundé, Cameroon	Assesses the state of MSW management and suggests possible solutions	▪ Lack of SWM infrastructure causes major negative health and economic impacts	▪ Environmental odor and pollution due to organic wastes	▪ Community support and involvement in MSW management ▪ Collaboration between agencies and nongovernmental organizations.
Dlamini et al. [44]	Johannesburg, South Africa	Reviews waste-to-energy technologies and their consequence on sustainable SWM	▪ Poor SWM practices cause health and social challenges	▪ Air and water pollution due to open-air burning, landfilling, and haphazard dumping and leachates	▪ Incineration technology for non-recyclable waste and anaerobic digestion of separated biodegradables for electricity generation. ▪ Increase resources, strong institutional and policy framework
Serge Kubanza & Simatele [49]	Johannesburg, South Africa	Evaluates solid waste governance in the city	▪ Threat to public health by emissions and leachates resulting from burning and burying of wastes	▪ Water, land, and air pollution due to waste burying, littering, and burning.	▪ Decentralize power to local communities with clear guidelines. ▪ More inclusive strategies that support public participation in SWM
Kabera & Nishimwe [13]	Kigali city, Rwanda	Analyzes the current state of MSWM.	▪ Adverse health impacts due to poor MSWM practices and infrastructure.	▪ Environmental pollution due to improper SW disposal.	▪ More state investment in SWM ▪ Create a relationship between local communities and private waste collectors. ▪ Mobilize private sectors to invest in SWM activities.
Muheirwe & Kihila [111]	Sub-Saharan Africa	Examines the current SWM regulation by exploring the global and national agendas.	▪ Negative impacts on human health due to poor enforcement strategies	▪ Environment degradation by pollutants due to poor SWM practices.	▪ Effective SWM policies, governance, and planning for resilient cities ▪ Participatory programs and effective political obligation.
Almazán-Casali & Sikra [50]	Liberia	Proposes an effective SWM system.	▪ Odor nuisance and GHG emissions via open-air burning are detrimental to human health.	▪ Land and water contamination via illegal burying and dumping of plastic and organic waste on land, trails, and waterways, ▪ Air pollution due to open-air burning	▪ Home-based waste separation. ▪ Reliable waste collection services within the neighborhood
Imam et al. [46]	Abuja, Nigeria	Develops an integrated and sustainable system for SWM in Abuja.	▪ Poorly engineered land disposal sites have compounded health-related issues.	▪ Environment degradation due to waste dumping along roads, beneath bridges, in drainage channels and culverts	▪ More effective private sector and informal sector integration and involvement. ▪ Composting and greater waste recycling and resource recovery
Mapira [47]	Masvingo, Zimbabwe	Assesses the current environmental challenges associated with SWM and disposal	▪ Negative impact on human health due to poor SWM practices.	▪ Environmental pollution and degradation due to indiscriminate waste disposal	▪ Environmental education and awareness campaigns in the community. ▪ Financial and technical assistance through donor aid
Adeleke et al. [108]	South Africa	Evaluates the trend, shortcomings, progress, and likely improvement areas for each sustainable waste management component	▪ A large quantity of unmanaged solid waste has detrimental effects on human health.	▪ Environmental quality deterioration because of poor SWM system.	▪ Intelligent waste management system modeling ▪ Intervention and involvement of multi-sectors
Muiruri & Karatu [79]	Eastleigh Nairobi County, Kenya	Assesses the household level solid waste disposal methods	▪ The proliferation of pathogens and associated disease-carrying vectors due to poor SWM practices ▪ Air and waterborne diseases	▪ Environmental risks include ecosystem degradation, soil and water contamination, global warming, and climate extremes.	▪ Promote public education and awareness campaigns about waste. ▪ Allocate more resources for effective SWM.
